# Supplementary material for: Exo84c interacts with VAP27 to regulate exocytotic compartment degradation and stigma senescence
Source: Nat Commun. 2023 Aug 14;14:4888. doi: 10.1038/s41467-023-40729-5 (PMC10425460; doi:10.1038/s41467-023-40729-5)
Supplement: Supplementary file 1 — Supplementary Information [file 41467_2023_40729_MOESM1_ESM.pdf]

Supplementary Information

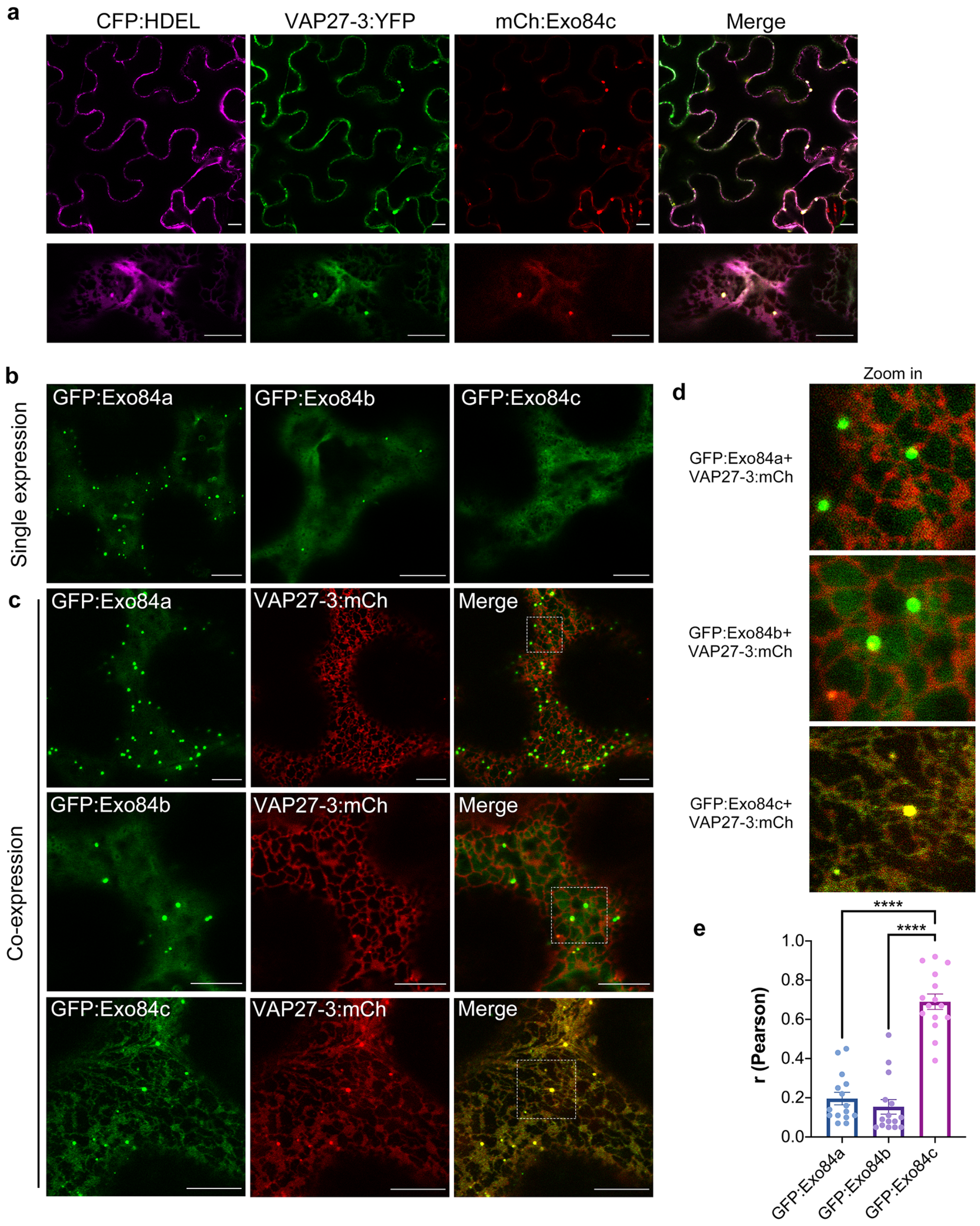

Supplementary Figure 1. Exo84c colocalises with VAP27 at punctate structures in *Nicotiana benthamiana*.

a The mCh:Exo84c and VAP27-3:YFP labelled punctate structures associated with CFP:HDEL-labelled ER network when transiently expressed together in *Nicotiana benthamiana*. Scale bars, 10 μm. The experiment was repeated twice with similar results. **b-d** VAP27-3:mCh transiently expressed with GFP:Exo84a, GFP:Exo84b or GFP:Exo84c in *Nicotiana benthamiana*. Transient single expression GFP:Exo84c mainly localizes at cytoplasm while GFP:Exo84a and GFP:Exo84b localizes cytoplasm and punctate structures (**b**). No colocalization is found between Exo84a/Exo84b and VAP27-3. GFP:Exo84c and VAP27-3:mCh colocalize at punctate ER derived structures (bottom panel, **c;** also seen in **d**). Scale bars, 10 μm. **e** The colocalization

between VAP27-3 and Exo84 isoforms (as in **b** and **c**) was quantified using the Pearson coefficient (n=15 cells). Error bars represent SEM, and an ANOVA analysis was employed for the quantification. The asterisks represent means that are significantly different at $P<0.05$, and a one-way ANOVA with Dunnett's multiple comparisons test was used for the analysis.

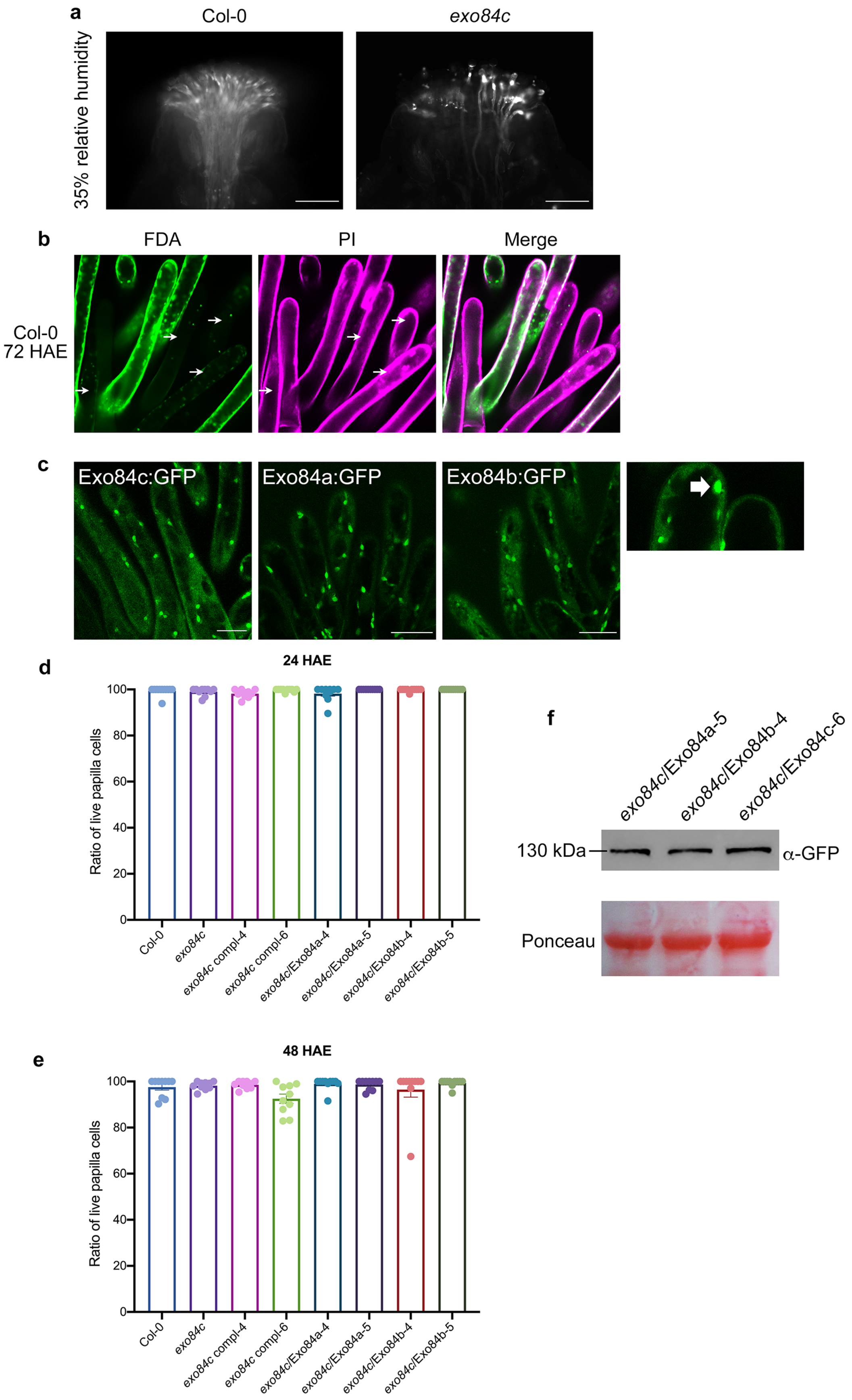

Supplementary Figure 2. Study the papillae senescence using FDA/PI dual staining.

a Less pollen grains germinated on exo84c mutant stigma than that on Col-0 stigma under low humidity. Scale bars, 100 μm. **b** Representative images of live/dead papilla cells in Col-0 plant at 72 HAE. **c** The localization of *exo84c*/Exo84cp::Exo84a:GFP, *exo84c*/Exo84cp::Exo84b:GFP, *exo84c*/Exo84cp::Exo84c:GFP and Sec6p::Sec6:GFP in stigmatic papilla cells. Scale bars, 20 μm. **d and e** Quantification of the live papilla cells from Col-0, *exo84c*, *exo84c* complemented plants (*exo84c*/Exo84cp::Exo84c:GFP, namely *exo84c* compl), *exo84c* complimented with Exo84cp::Exo84a:GFP and *exo84c* complimented with Exo84cp::Exo84b:GFP plants at 24 **d** and 48 HAE **e**. Ten stigmas from three independent plants were used for the quantification (n = 10). Error bars represent SEM, and an ANOVA analysis was employed for the quantification. **f** Immunoblot analysis of the expression levels of Exo84:GFP from *exo84c*/Exo84cp::Exo84a:GFP, *exo84c*/Exo84cp::Exo84b:GFP and *exo84c*/Exo84cp::Exo84c:GFP plants, which were used in **c**-**e**. Protein extracts from 10-d-old seedlings and a GFP specific antibody were used; the levels of different Exo84:GFP isoforms were similar. Ponceau staining is used to show equivalent loading in the three lanes.

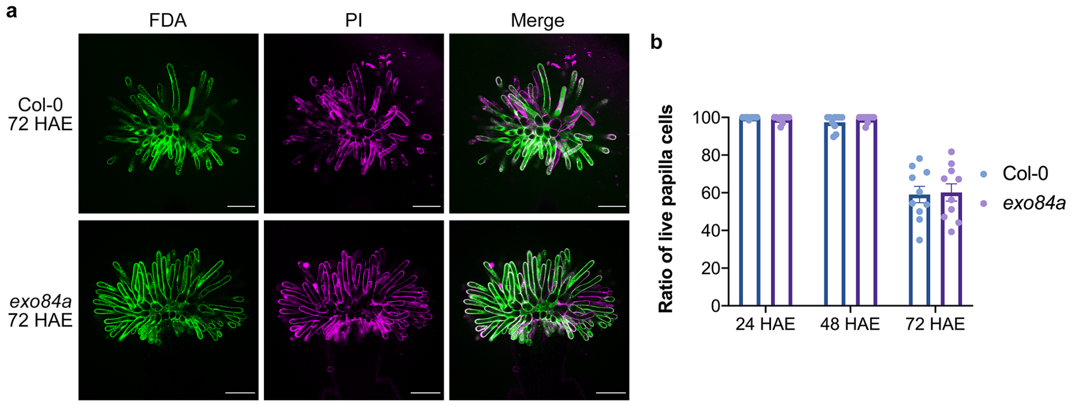

Supplementary Figure 3. Study the papillae senescence in the *exo84a* mutant using FDA/PI dual staining.

a Representative images of live/dead papilla cells in Col-0 plant at 72 HAE. Scale bars, 20 μm. **b** Quantification of the live papilla cells from Col-0 and *exo84a* plants at 24, 48 and 72 HAE. Ten stigmas from three independent plants were used for the quantification (n = 10). Error bars represent SEM, and an ANOVA analysis was employed for the quantification.

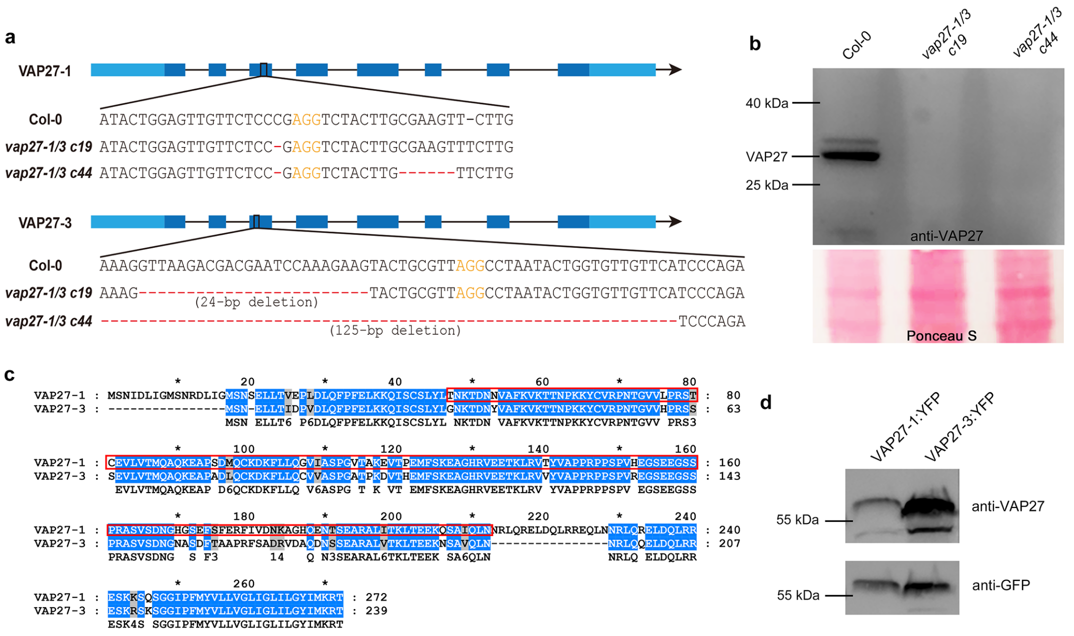

Supplementary Figure 4. The confirmation of *vap27-1/3* CRISPR mutant.

a Schematic diagram of *VAP27-1* and *VAP27-3* genes. The sequences of selected targets are shown below the diagram, respectively. The protospacer adjacent motif (PAM) sites are highlighted in yellow. Two homozygous double mutants, *vap27-1/3 c19* and *vap27-1/3 c44*, were obtained with deletions/insertions at the target sites. **b** Immunoblot analysis of Col-0, *vap27-1/3 c19* and *vap27-1/3 c44* using proteins extracted from 10-d-old seedlings and VAP27-1/-3 specific antibody, showing the knockout of VAP27-1 and VAP27-3 in the double mutants. Ponceau staining is used as the loading control. The experiment was repeated twice with similar results. **c** VAP27 antibody was raised against the peptide highlighted by the red box. **d** Both the VAP27 and GFP antibodies can recognize VAP27-1:YFP and VAP27-3:YFP which are transiently expressed in *N. benthamiana* of the same molecular weight, confirming the effectiveness of the antibody. The experiment was repeated twice with similar results.

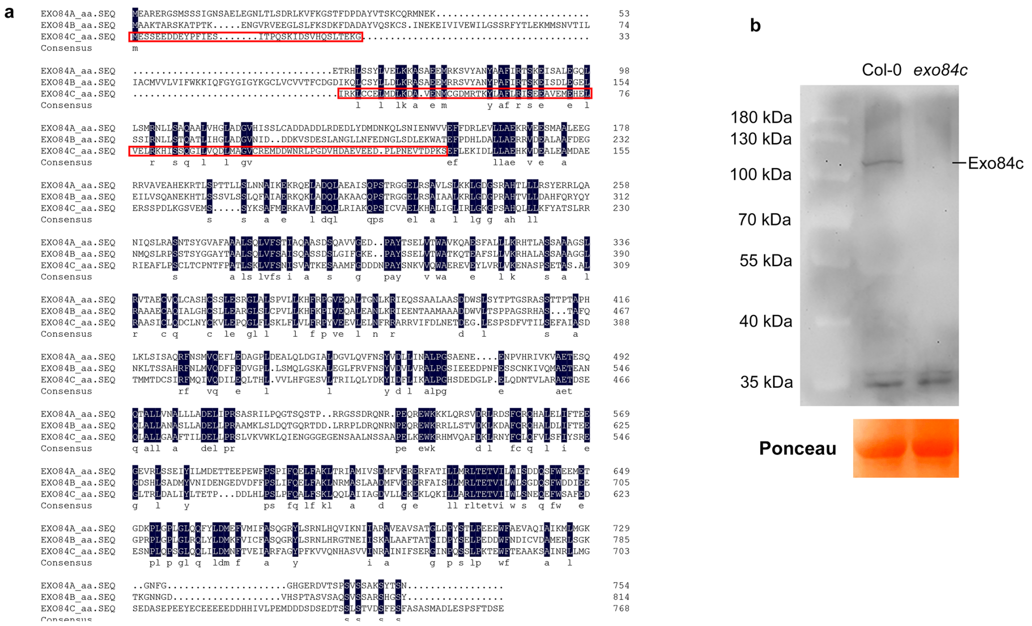

Supplementary Figure 5. The confirmation of Exo84c antibody.

a Exo84c antibody was raised against an Exo84c-specific peptide. **b** The antibody detects a band at approximately 100 kDa on the immunoblot of the Col-0 extract which is absent in the *exo84c* mutant extract. Ponceau staining is used to show equivalent sample loading. The experiment was repeated twice with similar results.

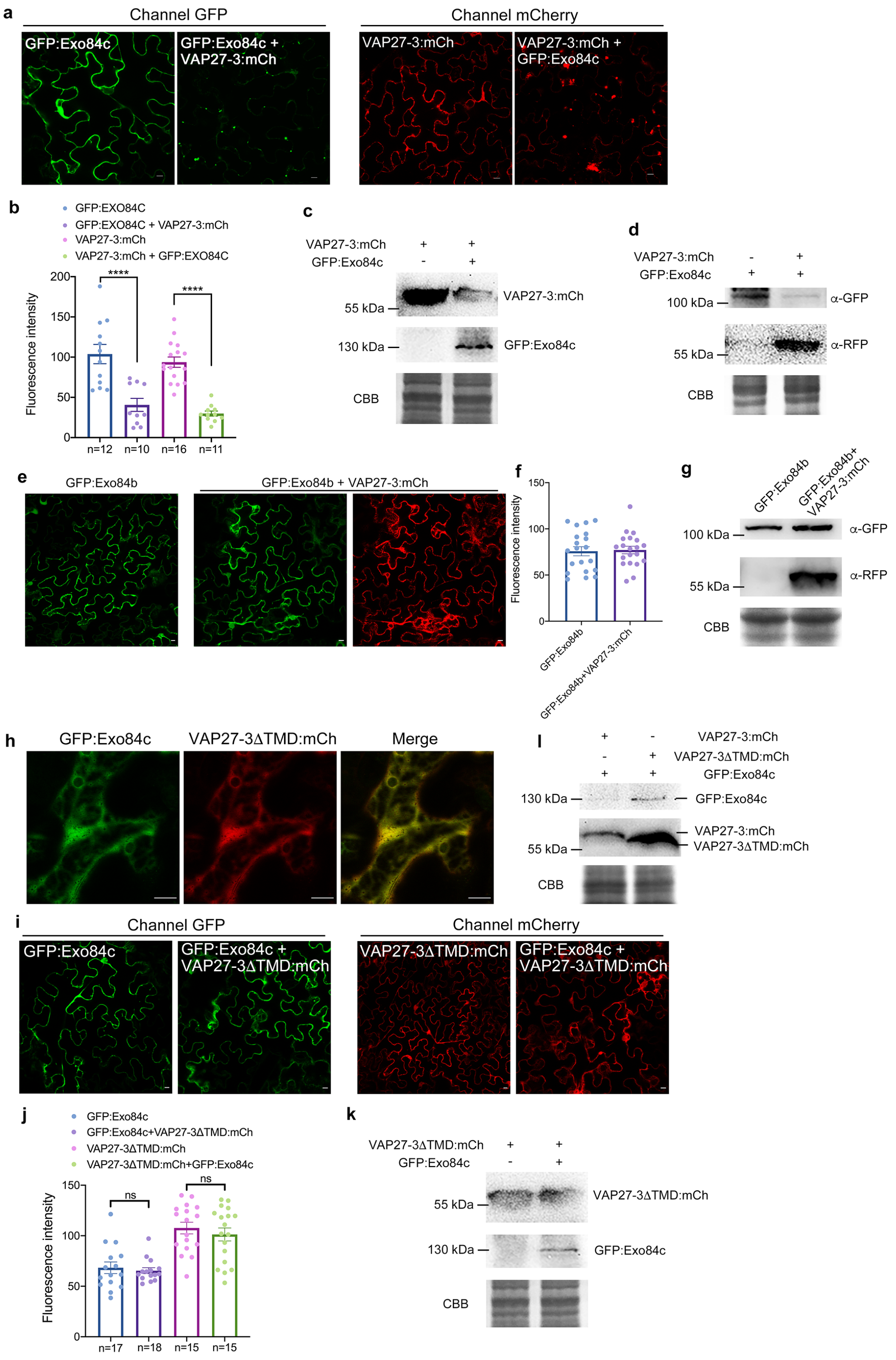

Supplementary Figure 6. Exo84c interacts with VAP27 and promotes their degradation, dependent on the ER-targetting domain of VAP27.

a Representative images of *N. benthamiana* cells transiently expressing GFP:Exo84c in the

presence/absence of VAP27-3:mCh; or transiently expressing VAP27-3:mCh in the presence/absence of GFP:Exo84c. Their protein abundancy was significantly reduced in co-expressed condition, judged from the signal intensity. Scale bars, 10 μm. **b** Quantification of the fluorescence intensity of Exo84c and VAP27-3 co-expressing cells in **a**, the fluorescence intensity of GFP:Exo84c and VAP27-3:mCh both significantly decrease. n represents the number of cells used for quantification. Error bars represent SEM, and the asterisks represent means that are significantly different at $P < 0.05$ from a one-way ANOVA with Tukey's multiple comparisons test. **c** Immunoblot analyses indicate that the level of VAP27:mCh is reduced in the presence of GFP:Exo84c, in agreement with the microscopy observation. CBB staining is used as the loading controls. **d** Immunoblot analyses indicate that the level of GFP:Exo84c is reduced in the presence of VAP27-3:mCh, in agreement with the microscopy observation. CBB staining is used as the loading controls. **e** Representative images of *N. benthamiana* cells transiently expressing GFP:Exo84b in the presence/absence of VAP27-3:mCh. Scale bars, 10 μm. **f** Quantification of the fluorescence intensity of Exo84b and VAP27-3 co-expressing cells in **a**, the fluorescence intensity of GFP:Exo84b does not change significantly (n = 20 cells). Error bars represent SEM, a two-tailed Student's *t*-test was used for the analysis. **g** Immunoblot analyses indicate that the level of GFP:Exo84b does not change significantly, in agreement with the microscopy observation. CBB staining is used as the loading controls. **h** VAP27-3ΔTMD:mCh (without the ER transmembrane domain) colocalizes with mCh:Exo84c only in the cytoplasm in *Nicotiana benthamiana*, no ER whorls are observed. Scale bars, 10 μm. **i** Representative images of *N. benthamiana* cells transiently expressing mCh:Exo84c in the presence/absence of VAP27-3ΔTMD:mCh, and cells transiently expressing VAP27-3ΔTMD:mCh in the presence/absence of mCh:Exo84c. Scale bars, 10 μm. **j** Quantification of the fluorescence intensity of mCh:Exo84c and VAP27-3ΔTMD:YFP in **i**. No significant differences are found. n represents the number of cells used for quantification. Error bars represent SEM, the asterisks represent means that are significantly different at $P<0.05$ from a two-way ANOVA with Sidak's multiple comparisons test. **k** Immunoblot analyses that indicate the level of VAP27-3ΔTMD:mCh remains at a similar level. **l** Immunoblot analyses of extracts of tissue samples expressing GFP:Exo84c with either full length VAP27-3 or VAP27-3ΔTMD. Coomassie brilliant blue (CBB) staining is used as the loading control.

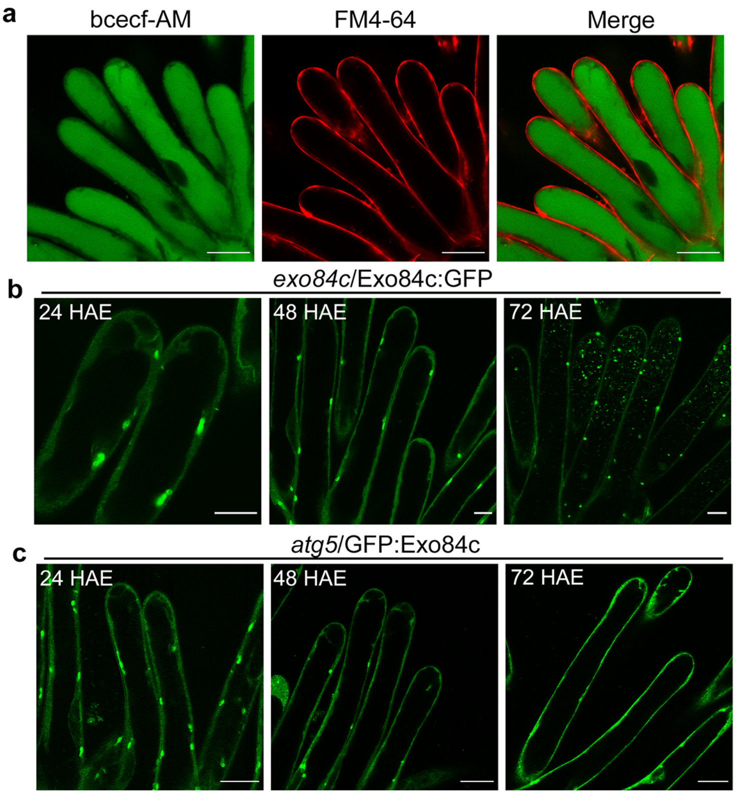

Supplementary Figure 7. Study the turnover of Exo84c and VAP27 in papillae cells.

a Papilla cells stained with FM4-64 (4 μM) and bcecf-AM (20 μM) to label the PM and vacuole, respectively. Scale bars, 20 μm. **b** Localization of Exo84c:GFP in stigmatic papilla cells at the endogenous expression level (*exo84c*/Exo84cp:: Exo84c:GFP) at 24, 28, 72 HAE. Scale bars, 20 μm. **c** The vacuole accumulation of Exo84c:GFP in papilla cells (as seen in b) was blocked in the *atg5* mutant. Scale bars, 20 μm. The experiments above were repeated twice with similar results.

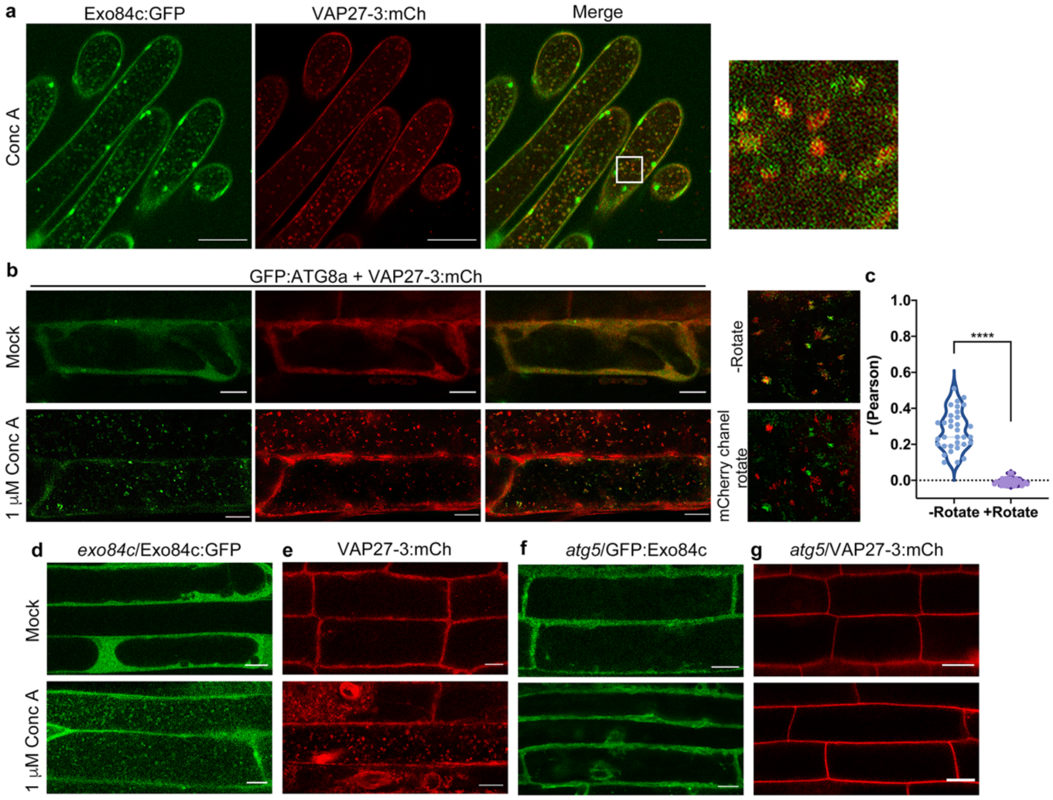

Supplementary Figure 8. Exo84c and VAP27-3 accumulate in the vacuole through autophagy.

a Papilla cells expressing Exo84cp::Exo84c:GFP and VAP27-3:mCh in the *exo84c* mutant background were treated with Conc A for 8 hours, and a strong accumulation of Exo84c and VAP27-3 co-labelled punctate structures were found in the vacuole. Scale bars, 20 μm. The experiment was repeated two times with similar results. **b** Root cells of 5-d-old Arabidopsis transformed with UBQ10::GFP:ATG8a + VAP27-3p::VAP27-3:mCh. After 1 μM Conc A treatment, numerous VAP27-ATG8 positive structures were found to accumulate in the vacuole. **c** The colocalization between VAP27 and ATG8a (as in **b**) was quantified using the Pearson coefficient (n = 33 cells). For the negative control, the mCherry channel was rotated by 90°, the significant difference was determined by a two-tailed Student's *t*-test, P<0.05. **d** At the endogenous expression level (*exo84c/*Exo84cp:: Exo84c:GFP), Exo84c accumulates in the vacuole following 1 μM Conc A treatment in root cells of stable transgenic Arabidopsis. **e** Stable Arabidopsis transformed with VAP27-3p::VAP27-3:mCh were treated with 1 μM Conc A. VAP27-3 labelled structures accumulate in the vacuole. **f and g** In the *atg5* mutant, the accumulation of Exo84c (**f**) or VAP27-3 (**g**) were blocked after Conc A treatment. Scale bars, 10 μm. The experiments in (**d-g**) were repeated twice with similar results.

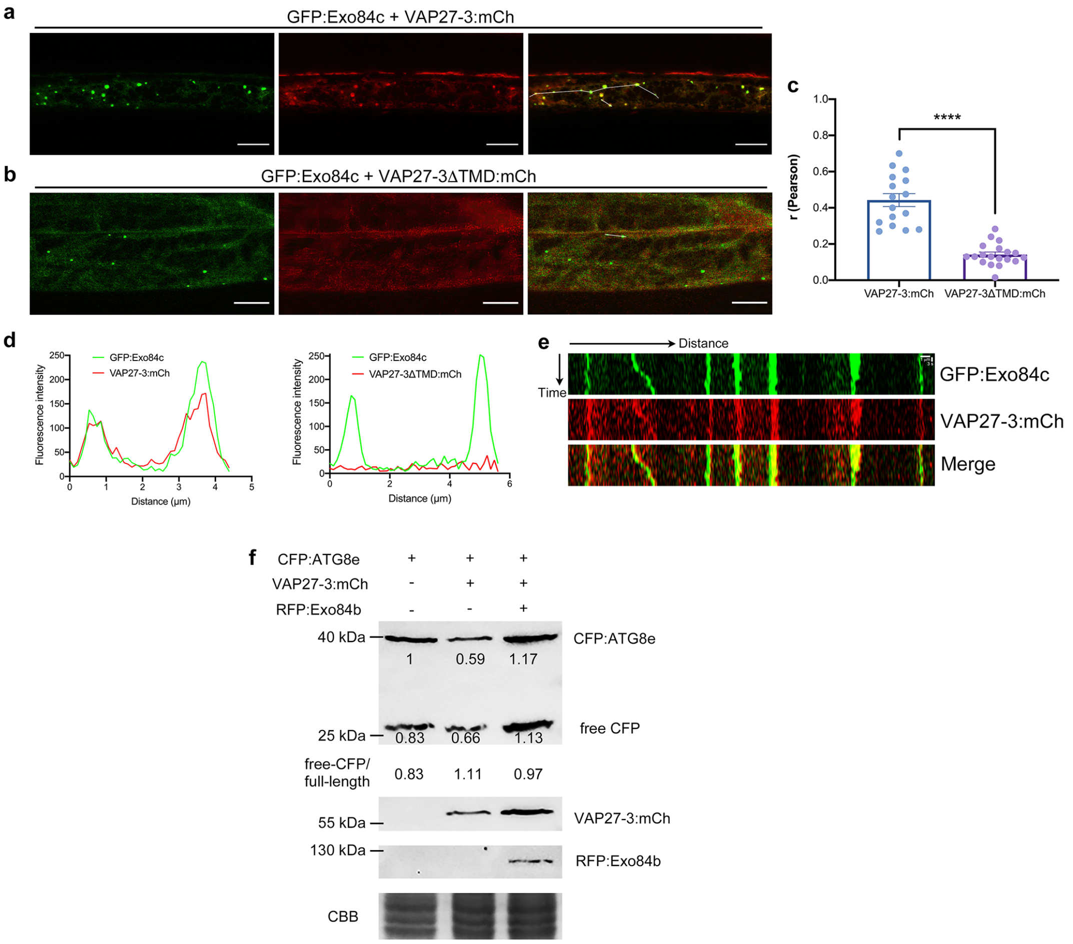

Supplementary Figure 9. Exo84c-labelled structures are closely associated with VAP27-3 labelled ER networks.

a and b Root cells of 5-d-old Arabidopsis transformed with UBQ10::GFP:Exo84c + VAP27-3p::VAP27-3:mCh **a** or UBQ10::GFP:Exo84c + UBQ10::VAP27-3ΔTMD:mCh **b**. Scale bars, 10 μm. **c** The colocalization between VAP27-3 and Exo84c (as in **a** and **b**) was quantified using the Pearson coefficient. 16 cells and 19 cells from Arabidopsis expressing UBQ10::GFP:Exo84c + VAP27-3p::VAP27-3:mCh or UBQ10::GFP:Exo84c + UBQ10::VAP27-3ΔTMD:mCh were used for quantification, respectively. Error bars represent SEM, the asterisks represent means that are significantly different at $P<0.05$ from a two-tailed Student's *t*-test. **d** Representative line traces of the fluorescence intensity from a cell transect in **a** and **b**, respectively. **e** Kymograph shows the spatiotemporal activation pattern of GFP:Exo84c and VAP27-3:mCh-labelled punctae in **a**. **f** Immunoblot analysis of the level of CFP:ATG8e when it is co-expressed with VAP27-3:mCh and RFP:Exo84b in *N. benthamiana* cells, as indicated by the accumulation in free CFP levels and ratio of free-CFP/full-length protein. CBB staining is used as the loading controls. The protein levels are normalized to the loading control.

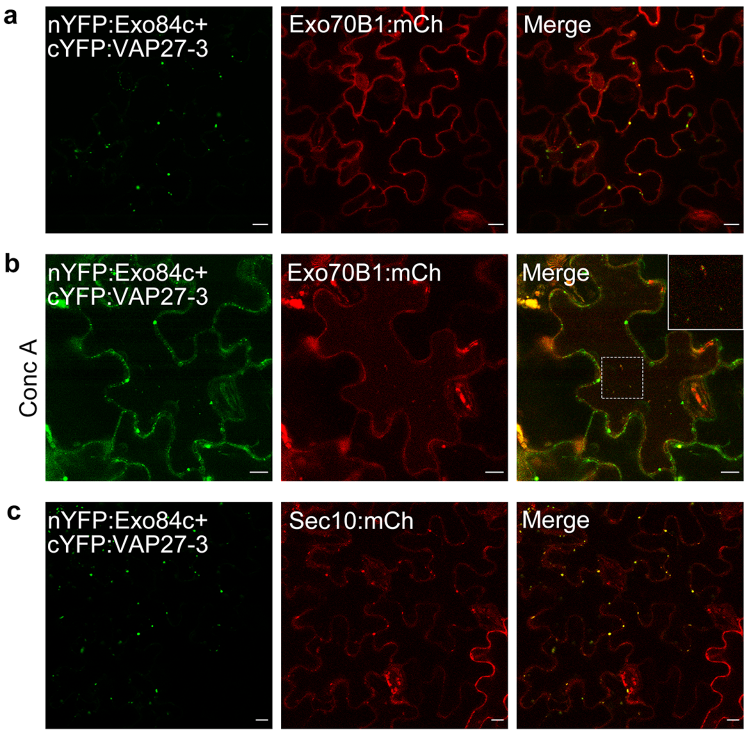

Supplementary Figure 10. The Exo84c-VAP27 labelled ER-autophagosome structures recruit several exocyst subunits in *Nicotiana benthamiana*.

a The exocyst subunit Exo70B1:mCh is recruited to the Exo84c-VAP27-3 labelled punctate structures in *N. benthamiana*. **b** Exo70B1:mCh accumulated in vacuole together with Exo84c and VAP27-3 upon Conc A treatment in *N. benthamiana*. **c** The exocyst subunit Sec10:mCh is recruited to the Exo84c-VAP27-3 labelled punctate structures in *N. benthamiana*. Scale bars, 10 μm. The experiments above were repeated twice with similar results.

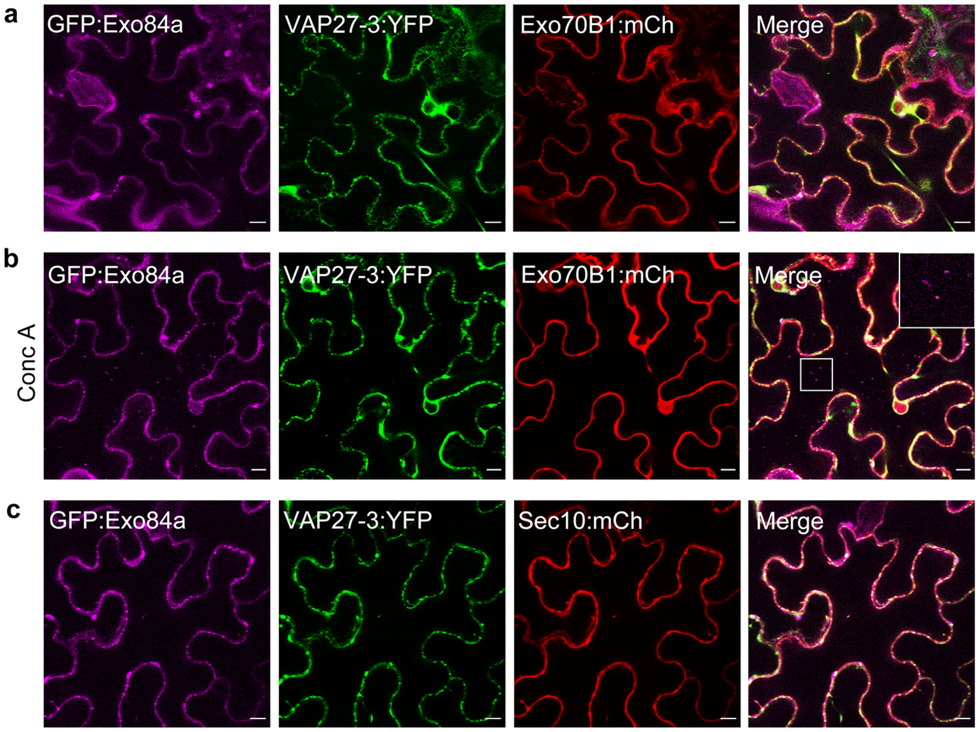

Supplementary Figure 11. Co-expression of Exo84a and VAP27 cannot recruit other exocyst subunits to puncta structures. a-c No colocalization is found when the exocyst subunits Exo70B1:mCh (**a**) or Sec10:mCh (**c**) is co-expressed with GFP:Exo84a + VAP27-3:YFP in *N. benthamiana*. Exo70B1:mCh did not accumulate in the vacuole together with Exo84a and VAP27-3 upon Conc A treatment in *N. benthamiana* (**b**). The experiments above were repeated twice with similar results. Scale bars, 10 μm.

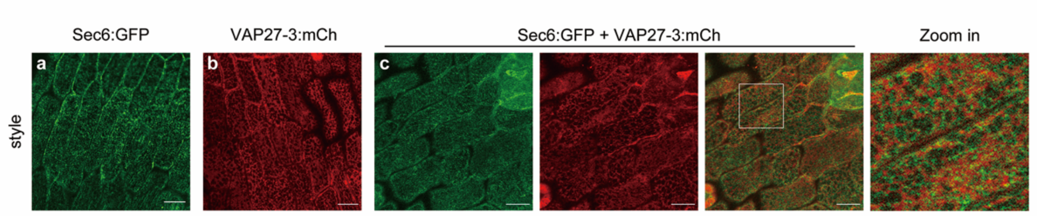

Supplementary Figure 12. Sec6-labelled exocytosis vesicles are closely associated with VAP27-3 labelled ER network in style cells of the pistil. a-c, Subcellular localization of Sec6 and VAP27-3 in Arabidopsis expressing Sec6p::Sec6:GFP (**a**), VAP27-3p::VAP27-3:mCh (**b**), and Sec6p::Sec6:GFP + VAP27-3p::VAP27-3:mCh (**c**) in style cells. The experiments above were repeated twice with similar results. Scale bars, 10 μm.

Supplementary Table 1. List of plasmids constructed in this research.

DNA fragment	Destination vector	Fusion protein
Exo84c full-length CDS	pMDC43	35S::mCherry:Exo84c
Exo84c full-length CDS	pUBN	UBQ10::GFP:Exo84c
Exo84cp::Exo84c	pMDC107	Exo84cp::Exo84c:GFP
VAP27-3p::VAP27-3	pMDC107	VAP27-3p::VAP27-3:mCherry
Sec10 CDS	pMDC83	Sec5a:mCherry
Sec6 CDS	Modified pUBC	Sec6p::Sec6:GFP
Exo70B1 CDS	pMDC83	Exo70B1:mCherry
Exo84b full-length CDS	pMDC43	mRFP:Exo84b
Exo84cp::Exo84b	pMDC107	Exo84cp::Exo84b:GFP
Exo84cp::Exo84a	pMDC107	Exo84cp::Exo84a:GFP
ATG8e full-length CDS	pK7WGC2	CFP:ATG8e

Supplementary Table 2. List of primers used in this study.

Primers	Sequences (5' - 3')	Usage
Exo84c-F<br>Exo84c-R	ATGGAGAGCAGCGAGGAAGA<br>TCAAGACTCAGAATCGGTGAAA	Exo84c full-length CDS
Exo84cp-F<br>Exo84cp-R	CCGCATACCCAGAAGCCGGTC<br>GTCTCTCGCTCTATCTATTTCCC	Exo84c promoter
VAP27-1P-F<br>VAP27-1P-R	ACGAACAAGACCGACAATAATG<br>GTTCAGTTGAATGGCAGACT	VAP27-1 peptide
Exo84cP-F<br>Exo84cP-R	ATGGAGAGCAGCGAGGAAGAC<br>AGACTTTGGATCGGTTACTTCATTAGGC	Exo84c peptide
Sec10-F<br>Sec10R	ATGACAGAAGGAATCAGAGCAAGA<br>GCTCAAGCTTGGCCACAAGG	Sec10 CDS
Sec6-F<br>Sec6-R	ATGATGGTCGAAGATCTTGGTGTG<br>AGTGAGTTTTCGCCACATAGATCC	Sec6 CDS
Sec6p-F<br>Sec6p-R	CAGTTTGTGGAGTATTTTAGAAAGAATTCG<br>CTTCGCTAAATCACCTTATTTACGAACTCC	Sec6 promoter
VAP27-6-F<br>VAP27-6-R	GCTAGGCTTTACCATACGTTAATG<br>TATCCGGTTCAATAAGTAGCCAATC	VAP27-6 genomic
Exo84b-F<br>Exo84b-R	ATGGCGGCGAAGACGGCCAG<br>TCAATAGCTGCCATGAGATCTCG	Exo84b full-length CDS
Exo70B1-F<br>Exo70B1-R	ATGGCGGAGAATGGTGAAGAG<br>TTTTCTTCCCGTGGTAGTCCCT	Exo70B1 CDS
Exo84a-F<br>Exo84a-R	ATGGAAGCGAGAGAAAGAGGATCAA<br>TCAATTACTGGTGTAAGATTTAGCAGAGGAAAC	Exo84a CDS
ATG8e-F<br>ATG8e-R	ATGAATAAAGGAAGCATCTTTAAGATG<br>TTAGATTGAAGAAGCACCGAATG	ATG8e full-length CDS
